# Anesthetic Management of a Pediatric Patient With Succinic Semialdehyde Dehydrogenase Deficiency Undergoing Dental Procedures

**DOI:** 10.1155/cria/3794766

**Published:** 2025-10-13

**Authors:** Zülfü Savaş

**Affiliations:** ^1^ Department of Anesthesiology, Dicle University Faculty of Dentistry, Diyarbakir, Türkiye, dicle.edu.tr

**Keywords:** anesthesia, gamma-aminobutyric acid, metabolic diseases, pediatric dentistry, succinic semialdehyde dehydrogenase deficiency

## Abstract

**Background:**

Succinic semialdehyde dehydrogenase deficiency is a rare autosomal recessive disorder of Gamma‐aminobutyric acid metabolism characterized by neurological deficits and an increased risk of seizures. Patients with succinic semialdehyde dehydrogenase deficiency may present unique challenges in anesthesia management due to altered neurotransmitter levels and possible drug sensitivities. The literature is sparse regarding perioperative management of these patients.

**Case Presentation:**

We present the case of a 7‐year‐old boy, who underwent dental procedures under general anesthesia, with genetically confirmed succinic semialdehyde dehydrogenase deficiency. Anesthesia was initiated by intravenous induction, and sevoflurane was used for maintenance, with careful use of intravenous midazolam and rocuronium. Local anesthetic (lidocaine with epinephrine) was administered. The procedure lasted 120 min and was uneventful. The patient recovered without complications and was discharged the same day.

**Conclusion:**

This case highlights the feasibility of sevoflurane‐based anesthesia in patients with succinic semialdehyde dehydrogenase deficiency. Safe perioperative management can be achieved with individual anesthetic planning, careful drug selection, and appropriate monitoring. Documentation of similar cases is necessary to establish evidence‐based anesthetic strategies in this rare patient population.

## 1. Introduction

Succinic semialdehyde dehydrogenase deficiency (SSADH‐D; OMIM #271980) is a rare disorder characterized by an autosomal recessively inherited GABA degradation disorder caused by mutations in the ALDH5A1 gene on chromosome 6p22.3 [1]. Transamination of GABA to succinic semialdehyde continues with its reduction to 4‐hydroxybutyric acid or hydroxybutyrate (GHB) in the absence of SSADH enzyme. GHB, which is an amino metabolite of GABA and a neuropharmacologically active compound, accumulates in the brain in this disorder and may have neurotoxic effects [2].

The clinical picture of SSADH‐D is quite heterogeneous. The fact that the symptoms are not prominent in most patients may lead to a delay in the diagnosis of the disease, especially in patients without a family history [3]. SSADH deficiency is characterized by a relatively nonprogressive encephalopathy characterized by mild ataxia, hypotonia associated with hyporeflexia and developmental delay in the first 2 years of life. Mental retardation and language delay are seen in most patients [4]. Attention deficit and hyperactivity, aggressive behaviors in early childhood, and anxiety and obsessive‐compulsive behaviors in adolescence and adulthood are more frequently observed [2, 5]. Seizures occur in about half of the patients, and generalized tonic‐clonic or atypical absence seizures are commonly seen [5]. Apart from the seizure phenotype, there is no progressive worsening of the disease. However, the highly variable clinical presentation of SSADH‐D and very poor genotype/phenotype correlation make the diagnosis difficult [6].

From an anesthetic perspective, many problems can arise in the management of patients with SSADH deficiency. Elevated levels of GABA and GHB may alter the pharmacodynamics of anesthetic agents that affect GABAergic transmission, particularly volatile anesthetics such as benzodiazepines, barbiturates, and sevoflurane. It may also lead to increased sensitivity to sedative drugs and increased risk of seizures in the perioperative period.

Despite these concerns, there are very few studies in the literature on anesthetic management in patients with SSADH deficiency. This case report aims to contribute to the limited body of knowledge by detailing the anesthetic management of a SSADH‐deficient pediatric patient undergoing dental treatments under general anesthesia.

## 2. Case Presentation

Written informed consent was obtained from the patient’s legal guardians for the publication of this case report.

A 7‐year‐old male patient with SSADH deficiency was scheduled for multiple extractions and restorations under general anesthesia due to limited cooperation and extensive dental caries. The disease was diagnosed by molecular genetic analysis showing a homozygous pathogenic mutation in the ALDH5A1 gene.

Our patient was followed up for hypotonia and developmental delay during the infancy after birth and was diagnosed with SSADH deficiency after genetic and metabolic tests. He was then followed up regularly by the pediatric metabolism and pediatric neurology departments. Preoperative EEG and brain MRI tests were performed at the age of 7.

His medical history was remarkable for generalized developmental delay, hypotonia, and mild ataxia. The patient had no history of seizures. Neurologic follow‐up was ongoing. Preoperative EEG was reported as normal and brain MRI showed foci with hyperintense signal changes in T2A and FLAIR in the white matter and subcortical areas at bilateral frontotemporoparietal levels. In the 24‐h urine amino acid levels panel, increased GABA, glutamine, glycine, histidine levels, and decreased aspartate, glutamate, methionine, and ornithine levels were remarkable.

The patient was evaluated preoperatively using a comprehensive multidisciplinary approach. He had no history of previous general anesthesia exposure or adverse reactions. Family history did not contribute. On physical examination, he was cooperative but cognitively delayed. His weight was 23 kg and height was 115 cm. Taking into consideration his comorbidities and general condition, the patient’s American Society of Anesthesiologists physical classification was Class III. Airway examination revealed no abnormalities. The results of preoperative laboratory tests including complete blood test, electrolytes, liver and renal function tests, and coagulation profile were within normal limits.

Standard ASA monitors (peripheral SpO_2_, ECG monitoring, and noninvasive blood pressure monitoring) were applied in the operating room. Before induction of anesthesia, intravenous access was obtained via peripheral venous access with a 22G angiocath. Despite the CNS findings on MRI, the absence of epileptiform activity on EEG guided the anesthetic plan to agents with anticonvulsant properties, such as sevoflurane and propofol. Firstly, low‐dose midazolam (0.05 mg/kg) was administered. Then, lidocaine (1 mg/kg), propofol (3 mg/kg), fentanyl (1 mcg/kg), and rocuronium (0.75 mg/kg) were used to facilitate endotracheal intubation. Furthermore, the patient was hypotonic; thus, neuromuscular blocking agents were titrated, and no premedication was administered. The patient was nasotracheally intubated with a number 5.0 cuffed spiral tube. Ondansetron (0.2 mg/kg) was administered to prevent postoperative nausea and vomiting. Anesthesia was maintained with sevoflurane in a 50% air/oxygen mixture. Neuromuscular monitoring (train of four [TOF] stimulation) could not be performed intraoperatively because the necessary devices were not available. Local infiltration of 2% lidocaine with epinephrine was used for pain control during dental procedures.

During surgery, intravenous fluid therapy was guided by body weight and maintenance requirements, and isotonic balanced crystalloid solutions (Plasma‐Lyte) were administered in according to Holliday–Segar Method (4‐2‐1 rule). The Holliday–Segar method refers to administration of 4 mL/kg/h for the first 10 kg of body weight, 2 mL/kg/h for the next 10 kg, and 1 mL/kg/h for each additional kilogram. Plasma glucose levels are regularly monitored. Given the potential for autonomic dysfunction in SSADH, both fluid overload and hypovolemia were avoided. Throughout the operation, normothermia was achieved using forced‐air warming, and intraoperative temperature was monitored with a nasopharyngeal probe.

The operative procedure lasted approximately 120 min. Hemodynamic measurements and respiratory parameters remained stable throughout the operation. No seizures, muscle rigidity, or autonomic instability were observed during surgery. At the end of the operation, paracetamol (10 mg/kg) was administered for postoperative pain prophylaxis, and sugammadex (4 mg/kg) was administered, and when adequate recovery was observed, the patient was extubated awake and transferred to the postanesthesia observation unit.

Postoperative recovery was uneventful. No signs of delayed awakening, agitation, or seizure activity were observed. After awakening, the patient was monitored for 4 h. Continuous pulse oximetry and regular neurological assessments were performed during the first hour. Intermittent monitoring was then performed until discharge.

The decision to discharge the patient the same day was based on the criteria of full consciousness, stable vital signs, absence of seizure activity, adequate spontaneous ventilation and airway control, and the ability to tolerate oral intake. Given the potential for delayed neurological findings in SSADH, these criteria were applied conservatively, and the patient met all criteria before discharge. The patient was discharged the same day with postoperative instructions.

## 3. Discussion

SSADH deficiency is a rare autosomal recessive inborn error of metabolism associated with a defect in GABA metabolism. Most SSADH patients will have a similar set of CNS symptoms such as hypotonia, developmental delay, cognitive impairment, seizures, and ataxia.

Perioperative care in patients with SSADH deficiency begins with a comprehensive preoperative examination and investigation of end organ involvement. Since CNS involvement is commonly seen in these patients, the current anticonvulsant treatment plan, if any, should be known and the severity and type of seizures should be learnt. Anticonvulsant drugs should be administered on the day of surgery to ensure that routine doses are not missed [7].

In general, the choice of anesthetic agent may have a limited impact on the perioperative care of patients with seizure disorders [8]. Inhalational and intravenous anesthetic agents, including barbiturates, propofol, and benzodiazepines, have strong anticonvulsant effects [9]. Although our patient had CNS involvement, he had no history of seizures and no history of anticonvulsant drug use. Therefore, except for midazolam, propofol, and sevoflurane with anticonvulsant effects used in general anesthesia, no other prophylactic anticonvulsant drugs were given.

Given the associated involvement of the CNS and the limited evidence‐based medicine resources on anesthetic management in these patients, no definitive recommendations can be made regarding the choice and safety of neuromuscular blocking agents to be used.

Dosage adjustment based on monitoring of the TOF is useful to guide redosing, document reversal, and avoid long recovery times. Neuromuscular blockade should be reversed at completion of the procedure, and full recovery should be seen before tracheal extubation. In patients with hypotonia, reversal of steroidal NMBAs (vecuronium or rocuronium) with sugammadex may be preferable, given its limited side‐effect profile and superior efficacy compared with cholinesterase inhibitors [10]. In our patient, we chose to use rocuronium and performed extubation with sugammadex prior to awakening after seeing muscle strength regained.

Significant CNS involvement with hypotonia and poor upper airway tone or control may predispose these patients to develop upper airway obstruction or respiratory failure in the postoperative period. These problems may be exacerbated by residual effects of anesthetic agents. Depending on the patient’s condition, the surgical procedure and the choice of anesthetic agent, postoperative monitoring of respiratory function may be necessary in selected patients. Our patient did not experience any significant respiratory distress in the postoperative period.

Pretreatment with intranasal drugs (dexmedetomidine or midazolam) or intramuscular ketamine may be required depending on the patient’s cognitive status and ability to adapt to care [11]. Given the patient’s compliance and the absence of anxiety or agitation, and the risk of paradoxical reactions (such as agitation, disinhibition, or respiratory failure) associated with benzodiazepines in SSADH deficiency, no sedative premedication was administered to avoid these potential side effects. This approach is consistent with the literature demonstrating increased GABAergic sensitivity in SSADH, where even standard doses of anxiolytics can elicit exaggerated or atypical responses.

While no perioperative complications were observed in our patient, patients with SSADH deficiency may encounter potentially critical intraoperative challenges. These may include autonomic instability with abrupt changes in blood pressure and heart rate, delayed emergence from anesthesia due to central nervous system hypersensitivity, or paradoxical reactions to sedatives such as benzodiazepines. Furthermore, postoperative respiratory failure may occur due to hypotonia, necessitating preparation for prolonged ventilatory support. Such adverse events should be anticipated and planned for in patients with SSADH deficiency.

To summarize, anesthetic considerations for these patients include maintaining antiseizure medications at home, maintaining behavioral medications at home, and using short‐acting anesthetic and neuromuscular blocking agents to reduce the risk of residual blockade and the potential for respiratory failure.

## 4. Conclusion

This case demonstrates that general anesthesia using sevoflurane and minimally responsive doses of sedative agents can be safely administered in pediatric patients with SSADH deficiency when individualized planning and careful monitoring are implemented. Based on this experience, several clinical lessons for future practice can be highlighted. Firstly, due to potential hypersensitivity associated with altered GABAergic activity, sedative agents should be administered at reduced doses of approximately 50% of standard pediatric doses. Secondly, continuous intraoperative neuromuscular monitoring is essential to guide dosing and ensure timely and safe reversal of muscle relaxants. Also, same‐day discharge is feasible but should be accompanied by prolonged postanesthesia observation and rigorous discharge criteria to detect delayed neurological recovery. Local anesthetics with epinephrine can be safely used under close hemodynamic monitoring.

Given the rarity of SSADH deficiency, detailed documentation of anesthesia protocols and outcomes in such patients is crucial to inform evidence‐based perioperative care guidelines.

## Consent

Written informed consent was obtained from the patient’s legal guardians.

## Patient Perspective

We were very anxious about the anesthesia due to our child’s rare condition. The nursing team explained everything in detail, and we are grateful for how they made sure the procedure went smoothly and without complications (Patient’s legal guardian).

## Disclosure

All authors approved the final version of the manuscript.

## Conflicts of Interest

The authors declare no conflicts of interest.

## Author Contributions

All authors contributed to the conception, writing, and critical revision of this manuscript.

## Funding

This research received no specific grant from any funding agency in the public, commercial, or not‐for‐profit sectors.

## Data Availability

The data supporting the findings of this study are available from the corresponding author upon reasonable request.
